# Immunohistochemical Evaluation of CD3, CD4, CD8, and CD20 in Decidual and Trophoblastic Tissue Specimens of Patients with Recurrent Pregnancy Loss

**DOI:** 10.3390/clinpract12020022

**Published:** 2022-02-28

**Authors:** Dimitrios Kavvadas, Sofia Karachrysafi, Pinelopi Anastasiadou, Asimoula Kavvada, Stella Fotiadou, Angeliki Papachristodoulou, Theodora Papamitsou, Antonia Sioga

**Affiliations:** 1 Laboratory of Histology and Embryology, Medical School, Aristotle University of Thessaloniki, 54124 Thessaloniki, Greece; kavvadas@auth.gr (D.K.); sofia_karachrysafi@outlook.com (S.K.); stellaff@auth.gr (S.F.); angeliquep@gmail.com (A.P.); sioga@auth.gr (A.S.); 2 Laboratory of Pathology, Department of Oral Medicine, Dental School, Aristotle University of Thessaloniki, 54124 Thessaloniki, Greece; apinelop@dent.auth.gr; 3 Biomedical Sciences Department, Faculty of Health Sciences, International Hellenic University, 57400 Thessaloniki, Greece; akavvada@auth.gr

**Keywords:** recurrent pregnancy loss, CD3, CD4, CD8, CD20, decidua, trophoblast

## Abstract

Recurrent miscarriages affect up to 5% of couples. CD3^+^ (T-lymphocytes), CD4^+^ (helper T-lymphocytes), CD8^+^ (cytotoxic T-lymphocytes), and CD20^+^ (B-lymphocytes) cells may participate in the pathophysiology of recurrent pregnancy loss (RPL). The aim of this study was to investigate the complicity of these molecules in RPL. The experimental specimens were obtained from 20 females who underwent miscarriages in the first gestational trimester, while the control group’s specimens consisted of 20 females who proceeded with voluntary pregnancy termination during the same period. Tissue samples were taken from the decidua basalis, decidua parietalis, and trophoblast (placental chorionic villi) and were studied using immunohistochemical methods. Monoclonal antibodies were used against CD3, CD4, CD8, and CD20 cells. The lymphocyte levels in the decidua parietalis displayed profound disparities among the two groups. The decidua basalis and trophoblast exhibited almost the same disparities regarding positive CD cells. The comparison of CD4^+^ and CD8^+^ cells in the endometrial tissue revealed a significant difference between the two groups of study. The analysis uncovered a strong relationship between RPL and the presence of CD3^+^, CD4^+^, CD8^+^, and CD20^+^ cells in the decidua parietalis tissue. The number of positive T cells was decreased in the decidual basalis and chorionic villi, proving that their absence significantly disrupts the balance of the immunological environment.

## 1. Introduction

Recurrent pregnancy loss (RPL) is described as the occurrence of two or three repeated abortions, prior to the 20th week of pregnancy [1]. The frequency of RPL is approximately 1–2%, affecting 5% of couples [1]. There are numerous risk factors, mainly metabolic and anatomic, accompanied by systematic abnormalities and infections [2,3]. To this date, even though there are a plethora of studies regarding the etiologic factors of RPL, the full comprehension of the immune dysregulation that causes miscarriages seems to be elusive [4].

Leukocytes are a significant component of the human endometrium of the female reproductive system. Their numbers increase to 10% of stromal cells, in the proliferative phase. Prior to the phase of implantation is the secretory phase, in which 20% of endometrial cells are leukocytes. In early pregnancy, the proportion of leukocytes increases to 30% of the decidual cells [5]. This indicates their implication in RPL [6,7,8,9].

CD4 (cluster of differentiation 4) cells are glycoproteins, which are located on the surface of immune cells, more specifically on the surface of Th (T-helper) cells, monocytes, macrophages, and dendritic cells [6]. CD8 (cluster of differentiation 8) cells are transmembrane glycoproteins, which function as coreceptors of the TCR (T-cell receptor) [6]. Class I proteins of the MHC have the capacity to bind CD8 cells and TCRs [6]. Darmochwal-Kolarz et al. [7] studied the variations in the lymphocyte phenotype of women with unexplained pregnancy losses in comparison with healthy ones. They found that the number of CD4^+^ T-lymphocytes and CD8^+^ T-cells was significantly higher in patients with recurrent pregnancy loss.

The CD3 antigen is expressed mostly in T-lymphocytes and less in other cells, e.g., Purkinje cells, macrophages, and Hodgkin and Reed–Sternberg cells. It is expressed not only in the early maturation of T-lymphocytes but also in the pre-thymocyte cortical stage and is located within the perinuclear space of the cells [8]. Mengyang Du et al. gathered blood samples from RPL cases and observed that, in the peripheral blood of these women, CD3^+^ T-cells were significantly increased in comparison with a control group consisting of women with a normal pregnancy. The risk of miscarriage was increased proportionally to the augmentation of women with the level of CD3^+^ cells. However, the miscarriage risk did not appear to further increase when the level of CD3^+^ T cells exceeded 67.84% [9].

CD20 is an antigen expressed in B-lymphocytes, particularly in mature B cells. It is not expressed in active plasmocytes [10]. CD20 is a non-glycosylated phosphoprotein, consisting of three hydrophobic regions that are embedded in the cellular membrane [10,11]. It is structured by two long ends—one amino and one carboxyl—which are located intracellularly, with only a portion of the antigen exposed extracellularly [10,11]. This protein has a variety of functions, such as the modulation of B-lymphocytes, proliferation, and differentiation. The CD20 molecule is expressed primarily during the development of pre-B-lymphocytes [11]. In an investigation undertaken with RPL patients, levels of CD45^+^, CD56^+^, CD16^+^, CD3^+^, CD8^+^, and CRTH2^+^ cells were similar to those in control groups. No significant difference in lymphocyte subset numbers or percentages was noted between patients whose later pregnancy was successful and those whose later pregnancy was unsuccessful [9].

In recent years, studies have attempted to figure out the relationship between regulatory T-cells and RPL cases. It is widely believed that these cells play a vital role in establishing and maintaining the maternal–fetal immune tolerance [12,13].

Taking into account that nearly 50% of RPL cases are still classified as unexplained [14], we performed this immunohistochemical study in order to contribute to the scientific community with more knowledge on the matter. Our hypothesis was based on the immunological profile of RPL. Thus, we studied the markers CD3, CD4, CD8, and CD20 and their implication in RPL. We expected a significant expression of immune cells in the endometrial tissues of women with recurrent miscarriages in the first trimester of gestation, compared to those with a normal early pregnancy until termination. Regardless of the comparative outcome, we consider whether or not specific cells contribute to the pathophysiology of unexplained recurrent miscarriages.

## 2. Materials and Methods

The miscarriage experimental group comprised 20 Caucasian Greek women, aged between 35 and 42 years, who miscarried during the first trimester of gestation. The control group consisted of 20 healthy Caucasian Greek women, aged between 27 and 39 years, who electively terminated their pregnancies during the first trimester of gestation. Informed consent was collected from all patients, and Ethical Committee approval was received. All 20 females from the experimental group (EG) underwent a very specific background check. They reported at least three miscarriages of unexplained etiology. Women with fewer than three miscarriages (even without an etiology, were excluded from the study). Additionally, several abnormalities and known causes of miscarriages were evaluated, in order for their exclusion (Table 1). Thus, the inclusion criteria constituted RPL cases with no known anatomical or systematic abnormality. The inclusion and exclusion parameters were further assessed through an explorative pathological examination. The study was conducted according to the guidelines of the Declaration of Helsinki and approved by the Institutional Review Board (or Bioethics Committee) of Aristotle University of Thessaloniki (School of Medicine) (Number of approval: 4-198/17-7-2019).

### 2.1. Pathology Examination

Tissue specimens were collected and prepared for the microscope. They were collected immediately after miscarriage or elective abortion and washed with distilled water for removal of mucus and blood. These specimens were placental endometrial tissue that was set to be discarded. Pathology examination of the tissues and immunohistochemical analysis was performed. Then, tissues were studied under a stereomicroscope, so that specimens from decidua, villus chorion and parts of the embryo could be distinguished and examined for formation abnormalities or placental lesions. Specimens with formation abnormalities or placental lesions were excluded from the study. In order to evaluate any morphological abnormality with the optical microscope, specimens were stained with eosin-haematoxylin. Specimens were collected from the distinguished decidua and the villus chorion. They were stabilized in aqueous solution of neutral formalin 10% *v*/*v* for 12–24 h. Subsequent to this, specimens were placed in an automatic machine for further processing, including fixation, dehydration, xylene clarification and paraffin embedding. Then, paraffin-embedded blocks of specimens were cut in 3 mm sections and transferred to positive-charged and properly prepared glass plates, which were kept in an oven at 37–40 °C for 30–45 min. Afterwards, specimens were stained with haematoxylin-eosin solution (Harris). Many specimens were excluded due to errors on the process of collecting the tissues and anatomical deficiencies. The stained specimens were examined with a microscope and the most suitable of them were selected for immunohistochemical study.

Furthermore, in order to perform immunohistochemistry, four antibodies were selected: CD3, CD4, CD8, and CD20. These antibodies were obtained from DAKO Products. The dilution for each antibody’s solution is 1:100 for CD3, 1:50 for CD4 and CD8, and 1:200 for CD20.

#### Immunohistochemistry

In all specimens, the sites of decidua basalis and trophoblast (chorionic villi) were identified using the cytokeratin antibody (CK7), which is positive in chorionic villi. Moreover, in order to distinguish the chorionic villi (trophoblast) from the decidual cells at the feto-maternal interface, duplicate sections were stained with a monoclonal antibody against prolactin, for the visualization of decidual cells. The unstained specimens were further processed using an automatic machine (Bond Max) that carried out the following standard procedures of our laboratory. First, deparaffinization was performed in xylene. Afterwards, specimens were immersed in absolute alcohol, in degressive densities 100%, 96% and 70% *v*/*v*, consecutively, and were rinsed with distilled water. Antigen retrieval was performed by incubation at various temperatures, depending on the antibody that was examined each time. Following this procedure, specimens were first rinsed with PBS buffer, then incubated in H_2_O_2_ for 5 min, to quench endogenous peroxidase activity and finally rinsed again with PBS buffer. Afterwards, specimens were covered with a solution of the primary tonic monoclonal antibody. These antibodies, as mentioned previously, are CD3, CD4, CD8, CD20 and were obtained from DAKO Products. The dilution for each antibody’s solution is 1:100 for CD3, 1:50 for CD4 and CD8, and 1:200 for CD20. Tonsil was used as an appropriate control. Eventually, specimens were washed using WAS solution. For the detection of immunohistochemical staining, specimens were firstly immersed in Post Primary solution. After being washed, specimens were immersed in polymer solution and then in chromogen diaminobenzidine (DAB) solution. Finally, specimens were stained with Haematoxylin–Eosin. Following the previous stages that were performed by the automatic processor, specimens were rinsed in tap water and dehydrated with escalating densities of ethanol solution (70, 96, and 100% *v*/*v*, consecutively) and xylene. Then, they were covered with tape, placed in glass plates and immersed in Canada balsam. After the immunohistochemical staining procedure, specimens were thoroughly examined.

### 2.2. Microscopic Evaluation

An optical ZeissTM microscope was used and photographs were taken using a ContaxTM camera, attached to the microscope. In total, endometrial specimens—from decidua basalis, trophoblast and decidua parietalis—were examined by 2 independent researchers, specialized in pathology evaluation. The intensity of staining was evaluated on a 4-scale measure. The evaluation of the intensities was performed on the main sites of our tissue, of which there were three: decidua basalis, trophoblast, and decidua parietalis. The examination was managed by two independent researchers, specialists in the field of pathology evaluation. The intensity of staining was qualitatively evaluated as negative (–), weak (+), moderate (++) and strong (+++) based on each researcher’s observations on the microscope. A distinct granular brown stain was scored as positive. According to several studies, to be considered as positive, specimens should present at least 5% of granular stains. Above that score, the evaluation is weak (5–20%), then moderate (20–60%), and finally, strong (consecutively, up to a score of >60%). This is a widely approved consensus among the pathologists for the staining in immunohistochemistry methods.

### 2.3. Statistical Analysis

The results were statistically analyzed and confirmed for their significance using the Mann–Whitney U test. The data were checked for normal distribution prior to the selection of the Mann–Whitney U test. Our two independent researchers evaluated the intensity of all specimens twice on a blinded process, randomly presented from a third party. The final findings of each observer were collated and differences were noted. Our findings on the staining were quite conspicuous, since the intensities of each researcher were rather similar. There were a few discrepancies, mostly among weak and moderate intensities, but they were exceeded.

Our distinguished data were divided into two independent groups, and Mann–Whitney’s null hypothesis was checked. The level of significance was at 0.05—two-tailed hypothesis—and the *p*-value was estimated for each study undertaken among the two groups. The null hypothesis asserts that the two samples are identical and do not differ significantly (*p*-value > 0.05), while the alternative hypothesis means that results differ significantly (*p*-value < 0.05). In order to evaluate each case study and perform the statistics, we used the SPSS Statistics software. Our results were double-checked using the Mann–Whitney Test Online Calculator (accessed on 5 May 2021). The photographic material that follows is merely an indicating proportion of the study that was performed.

## 3. Results

Both groups presented with a similar estimated gestational age at the time of the abortion (median age: 8 weeks). Each antibody was studied separately and, based on the statistics, conclusions were extracted. The expression of immune cells was determined as weak (5–20%), moderate (20–60%), and strong (>60%).

### 3.1. CD3^+^ Intensity of Staining

**Figure 1:**
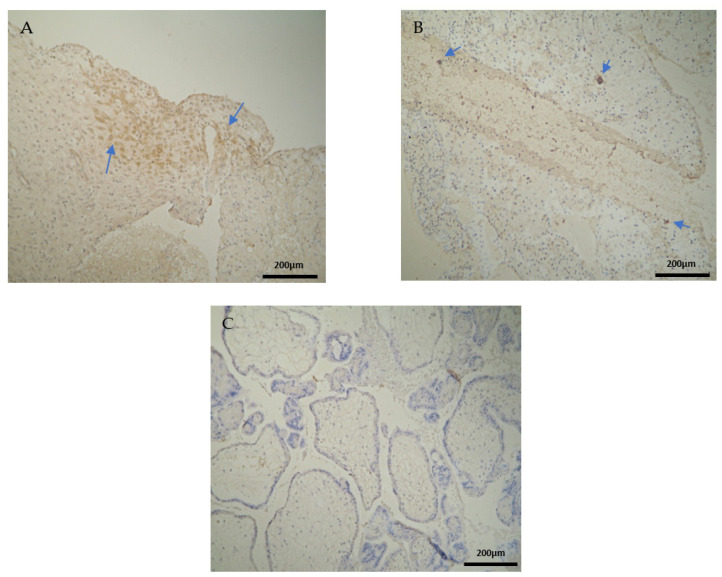
Evaluation of positive CD3 antigen expression. Miscarriage Experimental Group (EG): (**A**) Moderate CD3^+^ expression in the decidua parietalis (4 × 10 magnification). (**B**) Moderate CD3 antigen expression in the decidua basalis (4 × 10 magnification). (**C**) Chorionic villi sample with negative CD3 antigen expression (4 × 10 magnification).

The CD3 antigen expression in the decidua parietalis (DP) of the miscarriage experimental group (EG) was moderate in all RPL cases (Figure 1A) (Table 2). This finding demonstrates an immunological activity in the decidual part lining the uterus. The CD3 antigen expression was weak to moderate in 15 decidua basalis (DB) specimens (Figure 1B). No CD3 antigen expression was observed in the chorionic villi of this group (Figure 1C) (Table 2, Table 3 and Table 4). 

**Figure 2:**
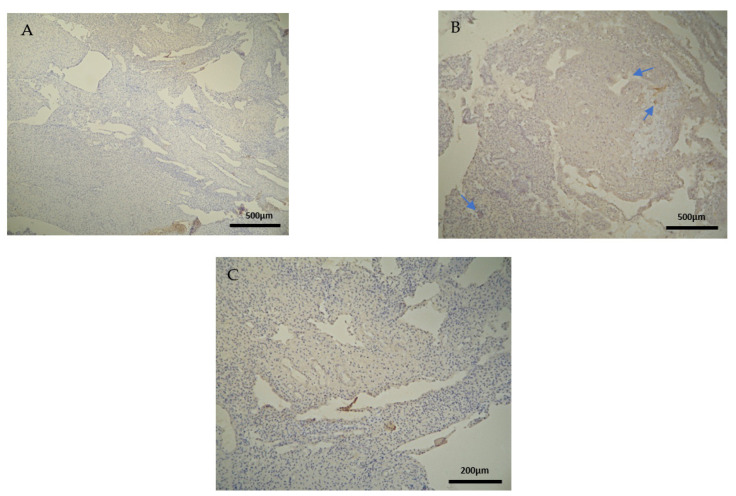
Evaluation of positive CD3 antigen expression. Control Group (CG): (**A**) there was no expression of CD3 in the DP (4 × 4 magnification). (**B**) There was moderate CD3 positive expression in the DB (4 × 4 magnification). (**C**) Decidua basalis and chorionic villi (4 × 10 magnification).

Negative CD3 antigen expression was observed in the decidua parietalis and chorionic villi of the control group (CG) (Figure 2A,C). Interestingly, the only CD3 positive expression was detected in the decidua basalis (Figure 2B). The part of the endometrium that participates with the chorion in the formation of the placenta exhibits the same immunological response between the control and the RPL cases (Table 3). 

### 3.2. CD4^+^ Intensity of Staining

**Figure 3:**
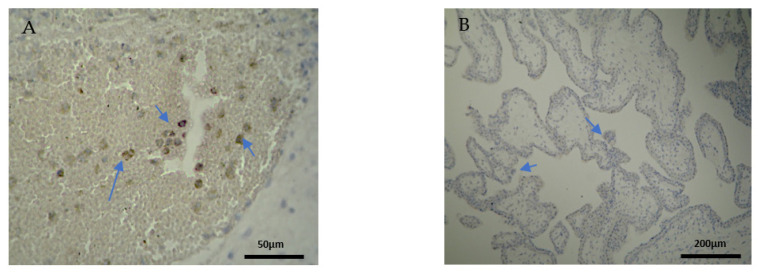
Evaluation of positive CD4 antigen expression. Miscarriage Experimental Group (EG): (**A**) Moderate intensity of staining in the DB (4 × 40 magnification). (**B**) Low intensity of staining in the chorionic villi (4 × 10 magnification).

The expression of the CD4^+^ antigen was weak to moderate in the DP, DB, and chorionic villi of the RPL cases (Figure 3A,B) (Table 2, Table 3 and Table 4).

**Figure 4:**
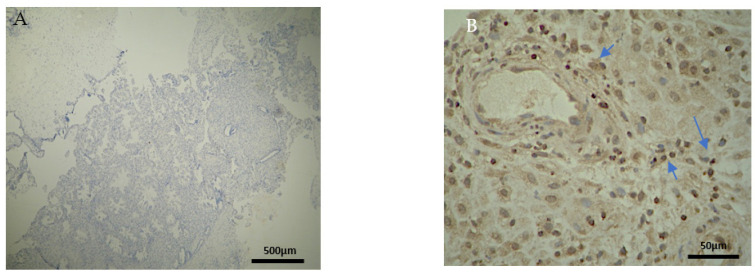
Evaluation of positive CD4 antigen expression. Control Group (CG): (**A**) No expression of CD4 in the DP (4 × 4 magnification). (**Β**) Moderate CD4 antigen expression in the DB (4 × 40 magnification).

The CD4^+^ antigen expression was negative in the DP of the electively abortion cases (CG) (Figure 4A) (Table 2). Surprisingly, the CD4 expression was positive in the DB and chorionic villi of the control group (Figure 4B) (Table 3 and Table 4). It seems that these specific villi that grow from the chorion to provide the maximal contact area with maternal blood are immunologically activated. Additionally, the fact that the expression in the decidua parietalis was significantly different in comparison to the rest of the sites (DB and trophoblast) among our RPL and control group was an unexpected finding (Figure 3 and Figure 4). 

### 3.3. CD8^+^ Intensity of Staining

**Figure 5:**
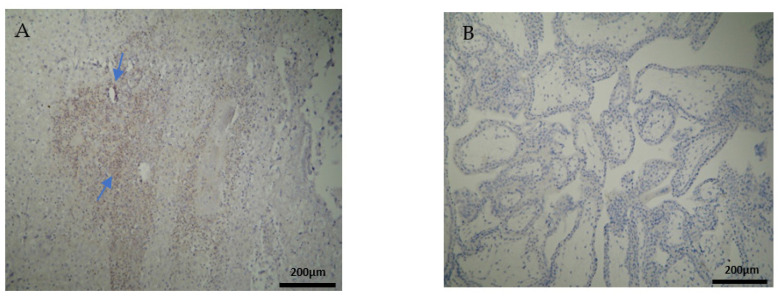
Evaluation of positive CD8 antigen expression. Miscarriage experimental group (EG): (**A**) Moderate expression of CD8 in the DP (4 × 10 magnification). (**Β**) Negative expression of the CD8 antigen in the chorionic villi (trophoblast) (4 × 10 magnification).

The CD8^+^ antigen expression in the decidua parietalis of the RPL specimens was low in 5 cases, moderate in 10 and strong in 5 cases (Figure 5A). Apart from the immunological expression, the positive CD8 cells that were found near the vessels revealed mild vascularization in the decidua parietalis. The decidua basalis and trophoblast were negative in all the RPL cases (Figure 5B) (Table 2, Table 3 and Table 4).

**Figure 6:**
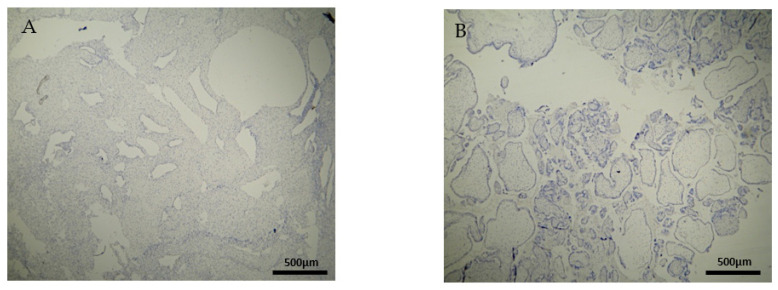
Evaluation of positive CD8 antigen expression. Control group (CG): (**A**) Negative CD8 expression in the decidua parietalis (4 × 4 magnification). (**Β**) Almost negative CD8 expression in the chorionic villi (trophoblast) (4 × 4 magnification).

Most of the electively abortion specimens were CD8 negative (Figure 6A,B) (Table 2, Table 3 and Table 4). The majority of the chorionic villi and DB specimens were marginally stained positive (Figure 6B, Table 3 and Table 4). A significant observation emerges by comparing Figure 5 and Figure 6; among the two groups, only RPL cases were positively CD8+stained and only in the decidua parietalis (Figure 5A, Table 2).

### 3.4. CD20^+^ Intensity of Staining

**Figure 7:**
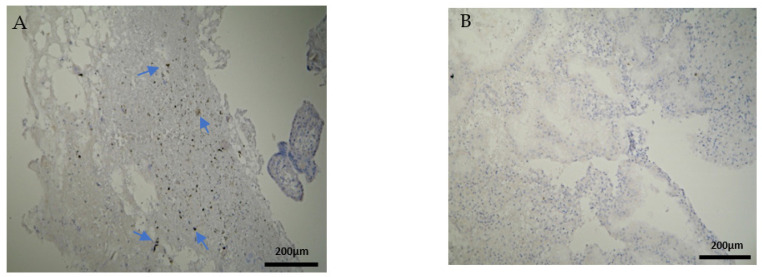
Evaluation of positive CD20 antigen expression. Miscarriage experimental group (EG): (**A**) Moderate CD20 intensity of staining in the DP (4 × 10 magnification). (**Β**) Negative expression of CD20 in the DB (4 × 10 magnification).

CD20^+^ antigen expression was almost similar to CD8^+^, as described above. More specifically, decidua parietalis was moderately stained in the majority of the RPL specimens (Figure 7A and Table 2). In the decidua basalis and trophoblast, CD20^+^ expression was negative in all the RPL specimens (Figure 7B and Table 3 and Table 4).

**Figure 8:**
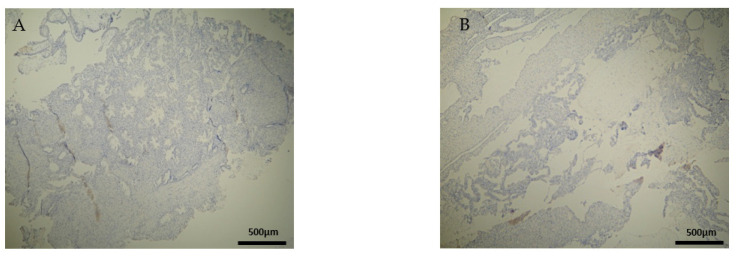
Evaluation of CD20 antigen positive expression. Control group (CG): (**A**) Negative CD20 expression in the DP (4 × 4 magnification). (**Β**) Negative CD20 expression in the DB (4 × 4 magnification).

Regarding the control group, the CD20 antigen expression was negative in all specimens (Figure 8) (Table 2, Table 3 and Table 4). Once more, in terms of antigens’ expression, significant differences were observed in the decidua parietalis of the RPL and control cases.

### 3.5. Tables and Schemes

**Figure 9:**
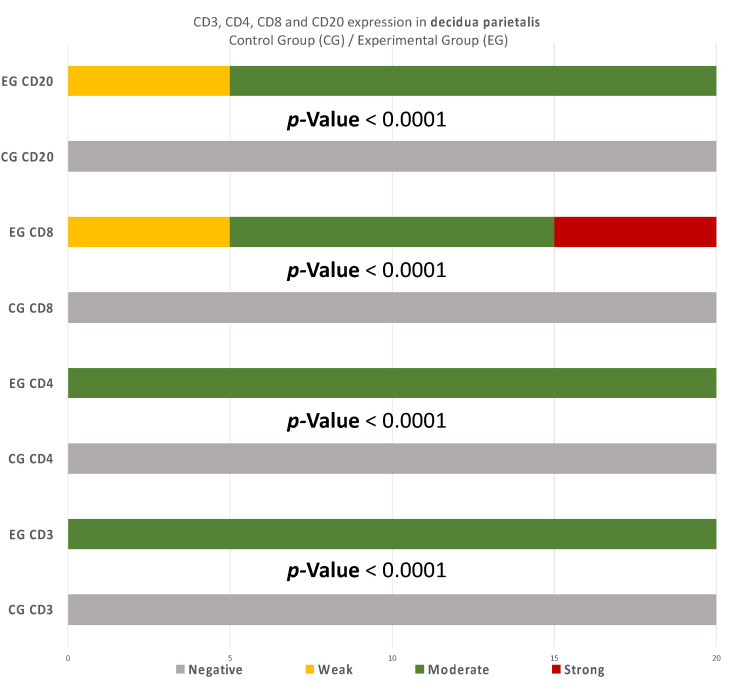
CD3, CD4, CD8, and CD20 antigen expression percentage in the decidua parietalis. The CG samples displayed negative CD3 and CD4 antigen expression, while the EG samples presented moderate CD3 and CD4 antigen expression. The CD8 antigen expression was negative in the CG specimens. Twenty-five percent of the EG samples presented weak CD8 antigen expression, while half of the group presented moderate and 25% strong expression. Finally, all the control samples were negative for CD20, while 25% of the EG samples were weak and 75% moderate. The result is significant at *p*-value < 0.05.

There were significant differences between the experimental and control group regarding the expression in the decidua parietalis (Figure 9) (Table 5).

**Figure 10:**
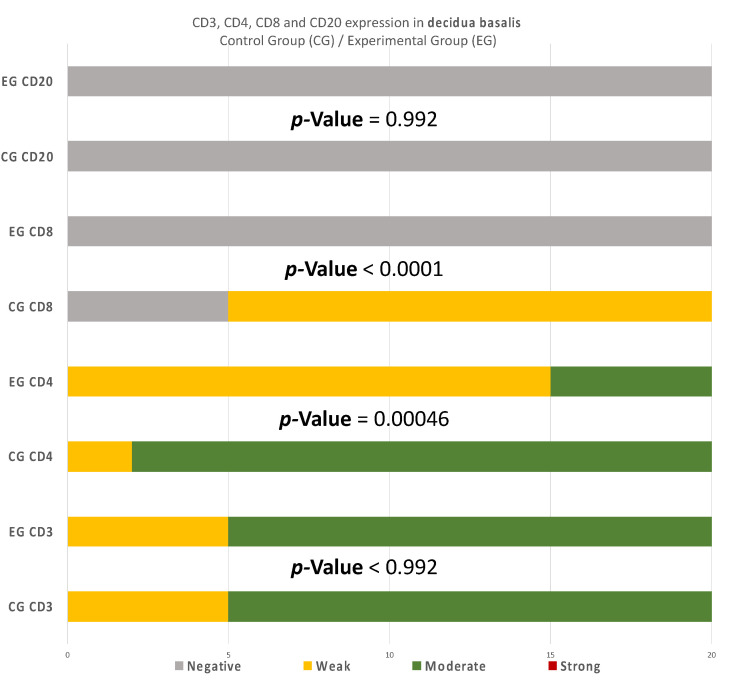
CD3, CD4, CD8, and CD20 antigen expression percentage in the decidua basalis. CD3 expression was similar in both groups. A total of 90% of the CG samples displayed a moderate CD4^+^ intensity while 25% of the EG samples were moderate and the rest were weak. All EG samples displayed negative CD8^+^ intensity. In contrast, 75% of the CG samples displayed of weak intensity. Finally, in regard to the CD20^+^ antigen expression, both groups were negative. The result is significant at *p*-value < 0.05.

There are also disparities in the decidua basalis among the miscarriage and control group (Table 3 and Table 4). B-lymphocytes cells detection appeared to be identical in the DB of the two groups (Figure 10).

**Figure 11:**
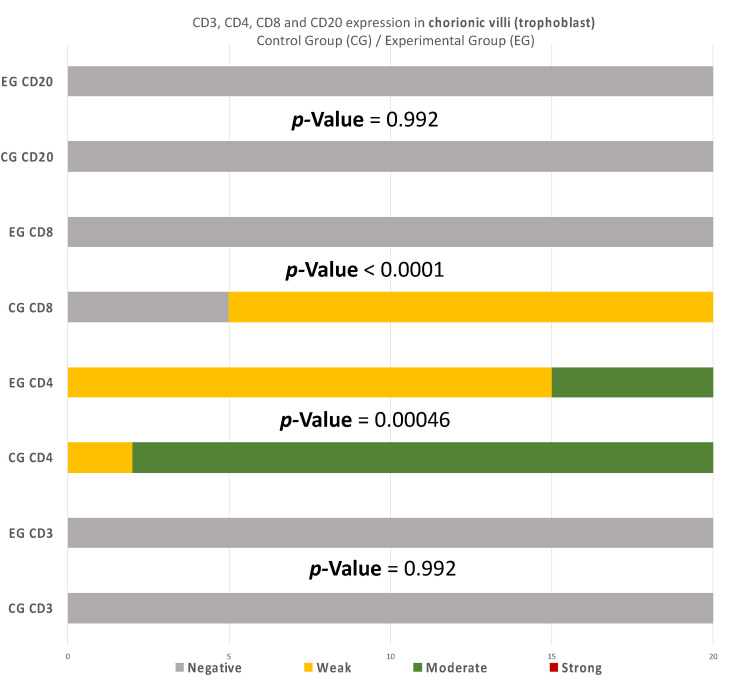
CD3, CD4, CD8, and CD20 antigen expression percentage in the chorionic villi (trophoblast). There was no expression of CD3 and CD20 in both EG and CG samples. Ninety percent of the CG specimens displayed moderate CD4 antigen expression, while EG specimens displayed mainly weak intensity. Finally, there was no positive CD8 expression in the EG samples. The result is significant at *p*-value < 0.05.

Similarly, in the chorionic villi of the RPL cases, the intensities of CD4^+^ and CD8^+^ were decreased in comparison to the control group (Table 4) (Figure 11). Additionally, no significant differences were observed. 

Summarizing the above analysis, statistically significant differences among the two groups were mainly found in the decidua parietalis (Figure 9). The decidua basalis and chorionic villi (trophoblast) displayed similar behavior regarding the expression of the studied markers, with CD4 and CD8 presenting considerable differences (Figure 10 and Figure 11).

More precisely, significant disparities were observed among the two groups regarding the expression of CD3 markers in the decidua parietalis, while in the DB and trophoblast, the results were the same (Table 5).

Regarding CD4 expression, there were significant differences among the two groups in every site of tissue examination (DP, DB, and trophoblast). It is worth mentioning that in the DP, the variation among the results was even more intense than the inconsistencies found in the DB and trophoblastic tissues (Table 6).

Furthermore, CD8 antigen expression displayed profound disparities among the two groups in the examined endometrial tissues (Table 7).

Finally, CD20 antigen expression appeared to be identical in the DB and trophoblastic cells of each group, while in the DP, the differences were quite significant (Table 8). It is noteworthy that the expression in the DP displayed inconstancies in every CD cell case of this study.

## 4. Discussion

The immunological environment of early pregnancy is rather complicated, especially between the RPL and abortion cases, which consisted of the experimental and control group of the present experiment. This study aimed to comparatively evaluate placental tissues received from the two distinguished groups. However, one should be cautious regarding the control group, as elective termination may not be able to provide a realistic depiction of the normal maternal-fetus environment. Regarding the immunohistochemical investigation, the present experiment made an attempt to correlate the imbalance of CD3, CD4, CD8, and CD20 levels, and the dysregulation of the immune system in the endometrial tissue, with the RPL pathogenesis (up to 80% of RPL cases) [15,16]. This study provides a comprehensive comparison of the CD3^+^, CD4^+^, CD8^+^, and CD20^+^ levels, in the decidua (both basalis and parietalis) and the chorionic villi, among women with RPL and women who electively terminated their pregnancy. Significant differences on the expression of the studied markers were found in the decidua parietalis (Table 2, Table 3 and Table 4) (Figure 9, Figure 10 and Figure 11).

To begin with, the miscarriage group samples presented a significantly higher expression in all the CD3 cells that were studied, in comparison to the control group samples (*p*-value < 0.00001) (Table 5). The moderate CD3 expression found in the EG is in agreement with Du et al. [9]. These results differ from Kolanska K et al. [17], who did not observe significant disparities on the CD3^+^ levels, among the experimental and control group. However, the fact that the present miscarriage group appeared to have a positive expression of CD3 in the decidua parietalis (Table 2) supports the hypothesis [9] that the risk of miscarriage phenomena increases proportionally to the augmentation of the CD3^+^ levels in the mothers’ peripheral blood. Given the current data—that maternal adaptive immune cells display a more activated phenotype in the decidua parietalis than the decidua basalis [18]—there is a strong indicator that increased CD3^+^ cells in the DP may contribute significantly to the occurrence of RPL phenomena. In addition, Quinn K et al. [19] has shown that the DP has a significantly higher ratio of T-regulator cells expression in comparison to the DB.

Likewise, there was a significant expression of CD4^+^ cells in the decidua parietalis of all RPL cases. The CG samples were negative (*p*-value < 0.00001) (Table 6). This particular finding arouses suspicion regarding the implication of CD3^+^ and CD4^+^ presence in the decidua parietalis tissues and their participation to RPL phenomena. There is suspicion that the strong CD4^+^ expression in the decidua parietalis is in accordance with the CD4^+^ positive serum levels in mothers’ peripheral blood, since they were observed to be particularly high in several RPL cases [7]. Contrary to the decidua parietalis, the expression of CD4^+^ in the decidua basalis and chorionic villi displayed an interesting variety of distribution. In most specimens, the control group demonstrated a moderate staining, while the experimental group appeared with low intensity (*p*-value = 0.00046) (Table 7). According to Li et al. [20] and Inada et al. [21], the percentage of CD4^+^ positive cells was lower in the decidua samples of early recurrent miscarriage patients, comparing to women with a normal early pregnancy. These interesting studies were in full accordance with the CD4^+^ levels found in the DB of our experimental and control group samples. This particular discovery leads us to the conclusion that T-cells in the decidua basalis and chorionic villi, are essential for a normal early pregnancy. In addition to that—according to Logiodice et al. [22]—CD4^+^ T-cells that were presented in the decidua of women who experienced a successful pregnancy were producing IL-4 (Interleukin), whereas in women with RPL, there was no IL-4 production. In the present study, we did not investigate the expression of CD25 for Treg-cells; thus, the aforementioned findings raise the question for further immunohistochemical analyses.

Furthermore, there were conspicuous disparities of CD8^+^ levels in the decidua parietalis among the two groups (*p*-value < 0.00001) (Table 7). One should definitely wonder whether the strong CD8^+^ antigen expression—similar to the DP of our EG samples—stands as an accomplice to the immune dysregulation, which leads to a potential pregnancy loss. The zero presence of CD8^+^ positive expression in the CG specimens strengthens the above hypothesis. According to further experiments, Darassejeze et al. [23] found that the prolonged activation of CD8^+^ cells was directly linked to the augmentation of miscarriage rates on rats. The aggregated positive expression near decidual vessels in our EG samples comes into full accordance with the conclusions of Russel et al. [24]. Despite these results, Lachapelle et al. [25] observed that the percentage of endometrial CD8^+^ T-Lymphocytes was significantly decreased in RPL cases. Our findings were quite similar to the aforementioned observation, since there was zero positive CD8 T-cells in both decidua basalis and chorionic villi of our EG samples. The control group’s results strengthen these findings, as a significant percentage of CD8^+^ T-cells was found, which is expected for a normal pregnancy to occur (*p*-value < 0.00001) (Table 7). Moreover, Johnsen G et al. [26] suggested that the absence of CD8^+^ in the decidua basalis, could be involved in further complications, such as the development of acute atherosis—a common spiral arterial lesion in preeclampsia. The analysis of the presence of CD8^+^ T-cells seems to be a rather controversial issue, as one could be led to contradictory results, depending on the site of study.

Recurrent miscarriages are associated by some studies with increased numbers of CD20^+^ lymphocytes [9,27]. In our experiment, an intense staining in the DP of the EG samples was found, contrary to the CG samples, where there was no expression. This antithesis establishes fundamental evidence of the strong relationship between RPL and the presence of CD3^+^, CD4^+^, CD8^+^, and CD20^+^ cells in the decidua parietalis (*p*-value < 0.00001) (Table 8).

### 4.1. Inconsistencies and Cohesions 

Based on prominent studies, there should not be any T- or B-cell expression observed in placenta tissues [28]. The present immunohistochemical study did not observe any expression regarding the studied markers in the decidua parietalis of the electively terminations (control group). However, the control group exhibited notable differences in the decidua basalis and trophoblast, which was not expected based on the aforementioned study [28]. There is an implication that some CD4 subsets minimize the embryotoxic state of other CD4 subsets; thus, it should be furthered studied [29]. Some other CD4 subsets initiate inflammatory processes through many cytokines. However, even in this case, there is a fragile balance that is crucial for placentation and early pregnancy [30]. For instance, even though TNF-a (induced by a CD4 subset) is the main culprit for several immunopathologic complications, it has been reported to have a protective role regarding the fetoplacental regulation [31]. Unfortunately, our study is limited to the subset analysis. It seems that CD4(+)CD25(+) Treg-cells are essential in the induction and maintenance of the essential immune-tolerance of normal pregnancy [32]. The CD4 levels in our study are intriguing and rather thought-provoking, especially regarding the control group. Additionally, several studies have shown that positive functional regulatory T-cells are decreased in the decidua basalis and chorionic villi of the RPL cases [12,33,34]. Our conclusive data were in full accordance to them and provided even more evidence regarding the CD^+^ levels in the DB and how they may affect the immunological regulation, which could lead to a miscarriage. The lower intensity of CD4^+^ and CD8^+^ in trophoblast and decidua basalis seems to negatively affect the course of a normal pregnancy, perhaps due to a significant reduction of the maternal immune tolerance. Regarding the CD20 cells, apart from the soaring levels in decidua parietalis, there was no significant difference in the decidua basalis and chorionic villi among the two groups. Positive T-cells were found in the decidua parietalis, displaying a more activated phenotype, with a higher intensity of staining, in comparison to those isolated from the DB and the trophoblast. This is a rather interesting outcome, as the decidua parietalis is not located in the maternal–fetal connective interface, and one would definitely not expect to find more activated T-cells there (at least not compared to the DB). This enacts as a proof of correlation among RPL cases and the increased levels of T-cells in the DP.

Finally, despite the contradiction, it should also be mentioned that there are several cases, e.g., Bohlmann et al. [35] and Michimata et al. [36], which did not observe a significant relationship between altered endometrial immunity and the RPL cases. Although the main culprits regarding immune dysregulation are known, the specific details that lead to RPL are still elusive. It is interesting that a study conducted among women with unexplained miscarriages and healthy fertile women with successful pregnancies revealed elevated CD4 lymphocytes in patients with recurrent pregnancy loss [7]. This study was not performed using electively terminations as controls; thus, despite the fact that we agree on the expression on RPL cases, it is not feasible to apply a proper comparison. Regardless of the methodological differences, each research is significant as it contributes on the elucidation of the immunological imbalance of early pregnancy. After all, despite so many remarkable studies, RPL remains an “unexplained” phenomenon to almost half of the cases [37].

### 4.2. Clinical Implications 

The clinical approach of the RPL depends on the pathogenesis of each condition. Chromosomal causes, anatomical abnormalities, clotting disorders, and hormonal and immunological causes must be investigated as a priority, as there are specific treatments for each condition. In order to understand the mechanisms of the unexplained RPL, researchers should collect as many data as possible regarding the immunological response when such phenomena occur. Thus, more experimental studies should be conducted. Immunization and maternal antibodies’ “behavior” might hold the key not only for setting a diagnosis but also for preventing pregnancy loss. There are several widely used and available algorithms assisting clinicians on the evaluation of each condition and offering possible explanation to parents suffering from miscarriages [38]. However, these algorithms come with painstakingly evaluation that put pressure not only on the couple but on the healthcare system as well. Diagnosis and possible treatment should be based on the causal factors of the RPL [38]. Therefore, clarifying the “picture” of the immunological reaction in the developmental tissue environment (e.g., decidua, trophoblast, etc.) might assist in the development of improved and rigorous algorithms for diagnosis and even new therapeutic interventions. 

### 4.3. Limitations and Strengths

This study has some limitation. As already mentioned, the percentage of the specific immune cells may be subjected to a margin of error due to the immunohistochemical evaluation of staining with a single antibody and not with a double-staining method. Another limitation is the lack of comparative laboratory methods (e.g., flow cytometry), in order to determine the specific cell levels on mothers’ peripheral blood, alongside the immunohistochemical placental evaluation. Generally, it seems that the immunological balance between mother and fetus is rather sensitive and volatile. Despite our exclusion criteria and the hypothesis (RPL vs. electively termination cases), there are many unknown factors that may affect the results. However, the study adds extra knowledge regarding the importance of distinguishing the sites of immunohistochemical evaluation, as the decidua parietalis presented conspicuous dissimilarities in comparison to the DB and trophoblast. Hopefully, with more studies, we will be able to narrow down the factors and utterly comprehend the process of immune dysregulation that leads to RPL.

## 5. Conclusions

In conclusion, in this study, we have performed an immunohistochemical experimental analysis of CD3, CD4, CD8, and CD20 markers on the decidual and trophoblastic tissue specimens of RPL cases and electively terminated pregnancies. The samples of the two groups differ significantly regarding the antigen expression levels. The uniqueness of the present experimental research lies not only in the studied markers, but also in the distinguish evaluation for each tissue site (decidua parietalis, decidua basalis, and chorionic villi). The analysis revealed a strong correlation among RPL and the presence of CD3^+^, CD4^+^, CD8^+^, and CD20^+^ cells in the decidua parietalis. Positive T-cells were decreased in the decidua basalis and chorionic villi of the RPL cases. This study suggests that the decidua basalis and decidua parietalis should be analyzed further by distinct immunohistochemical evaluation in each tissue site, as there is a strong indication that they do not act collectively regarding the immunological regulation during pregnancy. After proper clinical evaluation and status classifications, further studies on women may elucidate the unexplained RPL and lead from the diagnosis to the prevention. 

## Figures and Tables

**Table 1:** Test results to exclude the main abnormalities and known pathogenesis of RPL.

Possible Abnormalities for RPL	Tests Results
Parental karyotypes	None balanced translocation was detected
Intrauterine structural abnormalities	No adhesions or other structural abnormalities were found (hysteroscopy)
Luteal Phase Deficiency	Normal Endometrial Biopsy
Infections	Negative cervical cultures
Endocrinal evaluation	Normal values (TSH, insulin resistance, serum prolactin level, antithyroid antibodies)
Systemic Lupus Erythematosus	Normal serum level of IgG and IgM anticardiolipin antibodiesNegative test result for lupus anticoagulant
Non-APS thrombophilias	Normal thromborhilia evaluation (Homocysteine, factor V Leiden, prothrombin promoter mutation, activated protein C resistance)

**Table 2:** CD3, CD4, CD8, and CD20 antigen expression intensity in the decidua parietalis.

Marker Expression	CG CD3	EG CD3	CG CD4	EG CD4	CG CD8	EG CD8	CG CD20	EG CD20
Negative (−)	20	0	20	0	20	0	20	0
Weak (+)	0	0	0	0	0	5	0	5
Moderate (++)	0	20	0	20	0	10	0	15
Strong (+++)	0	0	0	0	0	5	0	0
SUM	20	20	20	20	20	20	20	20

CG: control group, EG: experimental group.

**Table 3:** CD3, CD4, CD8, and CD20 antigen expression intensity in the decidua basalis.

Marker Expression	CG CD3	EG CD3	CG CD4	EG CD4	CG CD8	EG CD8	CG CD20	EG CD20
Negative (−)	0	0	0	0	5	20	20	20
Weak (+)	5	5	2	15	15	0	0	0
Moderate (++)	15	15	18	5	0	0	0	0
Strong (+++)	0	0	0	0	0	0	0	0
SUM	20	20	20	20	20	20	20	20

CG: control group, EG: experimental group.

**Table 4:** CD3, CD4, CD8, and CD20 antigen expression intensity in the chorionic villi (trophoblast).

Marker Expression	CG CD3	EG CD3	CG CD4	EG CD4	CG CD8	EG CD8	CG CD20	EG CD20
Negative (−)	20	20	0	0	5	20	20	20
Weak (+)	0	0	2	15	15	0	0	0
Moderate (++)	0	0	18	5	0	0	0	0
Strong (+++)	0	0	0	0	0	0	0	0
SUM	20	20	20	20	20	20	20	20

CG: control group, EG: experimental group.

**Table 5:** CD3^+^ comparison among the experimental (RPL) and control (electively abortion) group.

Site of Examination	U-Value	Z-Test	*p*-Value	Result
Decidua parietalis	0	−5.4	<0.00001	significant
Decidua basalis	200	0.0135	0.992	not significant
Trophoblast	200	0.0135	0.992	not significant

The result is significant at *p*-value < 0.05 and the critical value of U at *p*-value < 0.05 is 127.

**Table 6:** CD4^+^ comparison among the experimental (RPL) and control (electively abortion) group.

Site of Examination	U-Value	Z-Test	*p*-Value	Result
Decidua parietalis	0	−5.4	<0.00001	significant
Decidua basalis	70	3.5	0.00046	significant
Trophoblast	70	3.5	0.00046	significant

The result is significant at *p*-value < 0.05 and the critical value of U at *p*-value < 0.05 is 127.

**Table 7:** CD8^+^ comparison among the experimental (RPL) and control (electively abortion) group.

Site of Examination	U-Value	Z-Test	*p*-Value	Result
Decidua parietalis	0	−5.4	<0.00001	significant
Decidua basalis	50	4.044	<0.00001	significant
Trophoblast	50	4.044	<0.00001	significant

The result is significant at *p*-value < 0.05 and the critical value of U at *p*-value < 0.05 is 127.

**Table 8:** CD20^+^ comparison among the experimental (RPL) and control (electively abortion) group.

Site of Examination	U-Value	Z-Test	*p*-Value	Result
Decidua parietalis	0	−5.4	<0.00001	significant
Decidua basalis	200	0.0135	0.992	not significant
Trophoblast	200	0.0135	0.992	not significant

The result is significant at *p*-value < 0.05 and the critical value of U at *p*-value < 0.05 is 127.
